# The Utility of Thermography in Detecting Subclinical Joint Inflammation at the Elbows in Patients With Rheumatoid Arthritis

**DOI:** 10.1111/1756-185X.70015

**Published:** 2024-12-16

**Authors:** York Kiat Tan, Rehena Sultana

**Affiliations:** ^1^ Department of Rheumatology and Immunology Singapore General Hospital Singapore Singapore; ^2^ Duke‐NUS Medical School Singapore Singapore; ^3^ Yong Loo Lin School of Medicine, National University of Singapore Singapore Singapore; ^4^ Centre for Quantitative Medicine, Duke‐NUS Medical School Singapore Singapore

Dear Editor,

Thermography, an emerging imaging modality, assesses joint inflammation by measuring joint surface temperatures objectively [1]. It has recently gained interest among researchers in the field of degenerative and inflammatory arthritides as evidenced by the increased publication trend in the past decade [2]. In comparison, ultrasound is an established tool for joint inflammation assessment, with its use in rheumatoid arthritis (RA) supported by a substantial amount of data that have amassed especially over the past two decades [3, 4]. According to the European Alliance of Associations for Rheumatology (EULAR) recommendations [4] and the American College of Rheumatology (ACR) guidance report [5], ultrasound may respectively be used to assess for persistent inflammation in patients with RA (as it can detect inflammation that predicts subsequent joint damage, even in clinical remission) [4] and can be reasonably used to evaluate for subclinical inflammatory arthritis in patients with mono‐ or oligoarthralgia without definitive diagnosis on clinical examination at certain specified asymptomatic joints or regions [5]. Although ultrasound can be a valuable tool for subclinical joint inflammation assessment in RA, its use is not without limitations [3]. For example, it can take considerable amount of training for sonographers to gain competency in musculoskeletal ultrasonography and scanning multiple different joint sites can be time‐consuming. Hence, there is a need to explore other low‐cost imaging modalities with high feasibility for use in the routine clinical practice for joint inflammation assessment. Thermography is safe, non‐invasive, with modern thermal cameras being compact, portable, and straightforward to use; hence thermography is well‐suited for use as an adjunctive tool in the busy rheumatology outpatient setting [1]. The elbow joint, important for daily activity, is chosen in our study as it is commonly affected in RA, with a 15‐year endpoint study revealing Larsen grade 2 radiographic erosion(s) in about two‐thirds of its RA study cohort [6]. In this single‐site cross‐sectional study conducted at a local tertiary hospital, we aim to compare thermography with ultrasound‐detected power Doppler (PD) and grey‐scale (GS) joint inflammation at clinically quiescent (non‐swollen; non‐tender) elbows of patients with RA fulfilling the 2010 EULAR/ACR RA classification criteria [7]. Our study conforms to the relevant research ethical guidelines and received approval by our local institutional review board. All patients provided informed consent before joining the study.

Clinical and imaging assessment at each patient's elbow occurred during the same study visit. Elbow joint swelling and tenderness were elicited by independent joint assessors (trained rheumatology nurses blinded to the imaging findings) as either yes/no. Ultrasound was performed by a rheumatologist with experience in musculoskeletal ultrasonography, whereas thermography was performed by another trained study team personnel while blinded to the ultrasound findings. Standardized ultrasonography following the EULAR guidelines [8] was performed using the Mindray M9 ultrasound machine (with a L14‐6Ns linear probe) with machine settings as follows: Doppler frequency, 5.7 MHz; pulse repetition frequency, 700 Hz. Ultrasound PD and GS synovial hypertrophy were scored semi‐quantitatively at the elbow's anterior humero‐radial and posterior fossa joint recesses using validated scoring methods [9]. Standardized thermography followed previously described methods [1, 10]. Patient was acclimatized by resting 15 min before starting thermal imaging, and thermography was performed in a windowless draft‐free room (ambient temperature around 24°C). A high performance thermal camera (FLIR T640) was used with the following settings: pixel resolution, 640 × 480; thermal sensitivity of < 30 m‐Kelvin at 30°C; predefined emissivity value of 0.98 for skin [1]. The elbow was imaged with the thermal camera placed 50 cm away from its anterior, posterior, lateral, and medial aspects. Using the commonly performed region of interest (ROI) manual segmentation approach [1, 10], the maximum, average, and minimum temperatures readings were obtained from the ROI at each elbow's aspect. The maximum, average, and minimum temperatures readings from the four aspects (anterior, posterior, lateral and medial) of the elbow were summed to obtained the total maximum (TMAX), total average (TAVG), and total minimum (TMIN) temperatures, respectively. The ultrasound PD and GS scores at the two joint recesses per elbow were summed to obtained the total PD score (TPDS) and total GS score (TGSS), respectively. Ultrasound synovitis at each joint recess is defined as PD ≥ 1 or GS ≥ 2 [3, 11]. Polyserial correlation and simple linear regression were used to study the relationship between thermographic (continuous) and ultrasound (ordinal) variables. Ability of TMAX, TAVG, and TMIN in predicting joint recess(es) with PD score ≥ 1 and GS score ≥ 2 were evaluated by calculating the area under the receiving operating characteristic curves (AUCs). Statistical analyses were performed using SAS version 9.4 software (SAS Institute; Cary, North Carolina, USA).

Our study included 35 right elbows from 35 RA patients with 140 elbow thermogram (four aspects per elbow) acquired and 70 joint recesses scanned by ultrasound. The baseline patients' characteristics were as follows: *n* = 26 (74.3%) female; *n* = 26 (74.3%) Chinese; mean (SD) age, DAS28, and disease duration were 57.9 (12.1) years, 3.67 (1.19), and 7.2 (6.4) months, respectively; all patients were on one or more of the following disease‐modifying anti‐rheumatic drugs (DMARDS): methotrexate, hydroxychloroquine, sulfasalazine, and leflunomide; *n* = 26 (74.3%) were on oral prednisolone. The TMAX, TAVG, and TMIN were significantly correlated (Table 1) with TPDS and the number of joint recess(es) with PD score ≥ 1 or GS score ≥ 2 (correlation coefficient ranging from 0.60 to 0.95, all *p* < 0.001). TGSS was significantly correlated with TAVG (correlation coefficient = 0.34, *p* = 0.036) but not with TMAX and TMIN (*p* > 0.05). Linear regression showed a statistically significant relationship (*p* < 0.05) between all the summed thermographic temperatures and ultrasound outcomes (TPDS, TGSS, and number of joint recess(es) with PD score ≥ 1 or GS score ≥ 2) with regression coefficient ranging from 0.08 to 0.15 (Table 2). The AUCs (95%CI) for TMAX, TAVG, and TMIN in predicting joint recess(es) with PD score ≥ 1 were 0.862 (0.701, 1.000), 0.848 (0.686, 1.000), and 0.822 (0.648, 0.996), respectively. The AUCs (95%CI) for TMAX, TAVG, and TMIN in predicting joint recess(es) with GS score ≥ 2 were 0.930 (0.830, 1.000), 0.880 (0.709, 1.000), and 0.833 (0.632, 1.000), respectively.

**TABLE 1:** Comparing thermographic parameters with ultrasound outcomes.

Thermo‐graphic parameter	TPDS			TGSS			Number of joint recess(es) with PD score ≥ 1 or GS score ≥ 2		
	Correlation coefficient	Standard error	*p*	Correlation coefficient	Standard error	*p*	Correlation coefficient	Standard error	*p*
TMAX	0.70	0.144	< 0.001***	0.31	0.166	0.064	0.95	0.049	< 0.001***
TAVG	0.64	0.156	< 0.001***	0.34	0.161	0.036*	0.83	0.136	< 0.001***
TMIN	0.60	0.169	< 0.001***	0.31	0.163	0.062	0.77	0.164	< 0.001***

Abbreviations: GS, grey‐scale; PD, power Doppler; TAVG, total average temperature; TGSS, total grey‐scale score; TMAX, total maximum temperature; TMIN, total minimum temperature; TPDS, total power Doppler score.

* *p* < 0.05.

*** *p* < 0.001.

**TABLE 2:** Linear regression between thermographic parameters and ultrasound outcomes.

Thermo‐graphic parameter	TPDS			TGSS			Number of joint recess(es) with PD score ≥ 1 or GS score ≥ 2		
	Regression coefficient	95% CI	*p*	Regression coefficient	95% CI	*p*	Regression coefficient	95% CI	*p*
TMAX	0.11	0.03, 0.18	0.007**	0.15	0.02, 0.28	0.028*	0.09	0.04, 0.15	0.002**
TAVG	0.09	0.02, 0.16	0.010*	0.15	0.03, 0.27	0.018*	0.09	0.04, 0.14	0.001**
TMIN	0.08	0.01, 0.16	0.021*	0.14	0.01, 0.26	0.031*	0.08	0.03, 0.14	0.003**

Abbreviations: GS, grey‐scale; PD, power Doppler; TAVG, total average temperature; TGSS, total grey‐scale score; TMAX, total maximum temperature; TMIN, total minimum temperature; TPDS, total power Doppler score.

* *p* < 0.05.

** *p* < 0.01.

To the best of our knowledge, our study is the first to demonstrate that thermographic temperatures are associated with ultrasound‐detected subclinical joint inflammation at clinically quiescent (non‐swollen; non‐tender) elbows of patients with RA. Thermographic temperatures showed good correlation with ultrasound PD joint inflammation and the number of joint recess(es) with ultrasound synovitis. In a separate study [12], histologic evidence of synovitis (including synovial lining cell hyperplasia, increased vascularity, and lymphocytic infiltrates) was observed in five out of six patients with RA and five out of eight patients with undifferentiated arthritis who underwent blind needle biopsies at their clinically asymptomatic knee joints. In an ultrasound study involving the bilateral wrists and finger joints (metacarpophalangeal joints (MCPJs) 1–5, thumbs' interphalangeal joints and the proximal interphalangeal joints 2–5) among 29 RA patients in clinical remission without physical synovitis, PD subclinical synovitis was associated with bone erosion at both the patient and joint levels [13]. In an magnetic resonance imaging (MRI) study involving the unilateral wrist, MCPJs 2–5 and metatarsophalangeal joints 1–5 among 113 early arthritis patients, clinically non‐swollen joints with subclinical inflammation (≥ 1 MRI‐inflammation feature) had an increased risk of radiographic progression within the first year [14]. In our study, thermography appears useful in predicting PD positivity and GS joint inflammation severity at the joint recess(es) of clinically quiescent (non‐swollen; non‐tender) elbows (with AUCs > 0.80). As thermography has high feasibility for use (e.g., low‐cost, simple and convenience to use) with less expertise and training required for its operators when compared to ultrasonography [1, 3, 15], further studies on its use as a first‐line screening tool for subclinical joint inflammation assessment at the elbows (and potentially at other joint sites) is warranted.

The main limitations of our study are the relatively small sample size and the cross‐sectional study design. Nonetheless, our correlation analysis of thermography with PD joint inflammation and the number of joint recess(es) with ultrasound synovitis appear robust and the results are unlikely to be due to chance as evidenced by the highly significant *p*‐values. Future larger scale RA studies with a prospective longitudinal design will be required to look at the sensitivity to change of thermographic parameters over time. Pertaining to how thermography compare with ultrasound, we did not proceed to look for cutoff value(s) nor perform the accompanying sensitivity and specificity analyses in view of our relatively small sample size (and correspondingly smaller number of ultrasound outcomes); future RA studies with a larger sample size will be required for a more robust analysis. Another limitation of our study is the absence of healthy controls. Future RA thermographic studies should ideally include healthy controls for comparison. As osteoarthritis can coexist in the same joint affected by RA, future well‐designed studies will need to look at how osteoarthritis may influence imaging outcomes at the elbow of patients with RA.

In conclusion, our study demonstrated that thermographic parameters are associated with ultrasound‐detected subclinical joint inflammation at clinically quiescent (non‐swollen; non‐tender) elbows of patients with RA; and appears useful in predicting PD positivity and GS joint inflammation severity at the elbow's joint recess(es). Thermography appears promising and our findings will need to be further validated in other independent RA cohorts.

## Author Contributions

Y.K.T. conceptualized the study and was involved in acquisition of data. R.S. performed the statistical analysis. All authors were involved in data interpretation, drafting, and preparation of the manuscript. All authors have approved the manuscript for publication.

## Conflicts of Interest

The authors declare no conflicts of interest.

## Data Availability

Data available upon reasonable request from the corresponding author.
